# Atypical presentation of barium reflux due to bronchoesophageal fistula

**DOI:** 10.1002/ccr3.3403

**Published:** 2020-10-12

**Authors:** Yeon Jae Han, Geun‐Young Park, Sun Im

**Affiliations:** ^1^ Department of Rehabilitation Medicine Bucheon St. Mary's Hospital College of Medicine The Catholic University of Korea Bucheon‐si Korea

**Keywords:** aspiration, bronchoesophageal fistula, esophageal cancer

## Abstract

Subtle signs of an abnormal communication from the esophagus to the respiratory tract space via the fistula may be missed and can result in delayed diagnosis. Our case shows an atypical presentation of a fistula during barium swallowing test.

## Graphical abstract

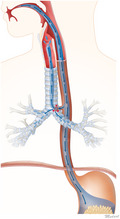

## 1 VIDEO CASE

A 67‐year‐old man with esophageal cancer (pT3N1M0, stage IIIA), who had recently been diagnosed with aspiration pneumonia, was referred for a barium swallow test. He had undergone the Ivor‐Lewis operation 10 months ago and had just completed concurrent chemoradiation therapy.

**FIGURE 1:**
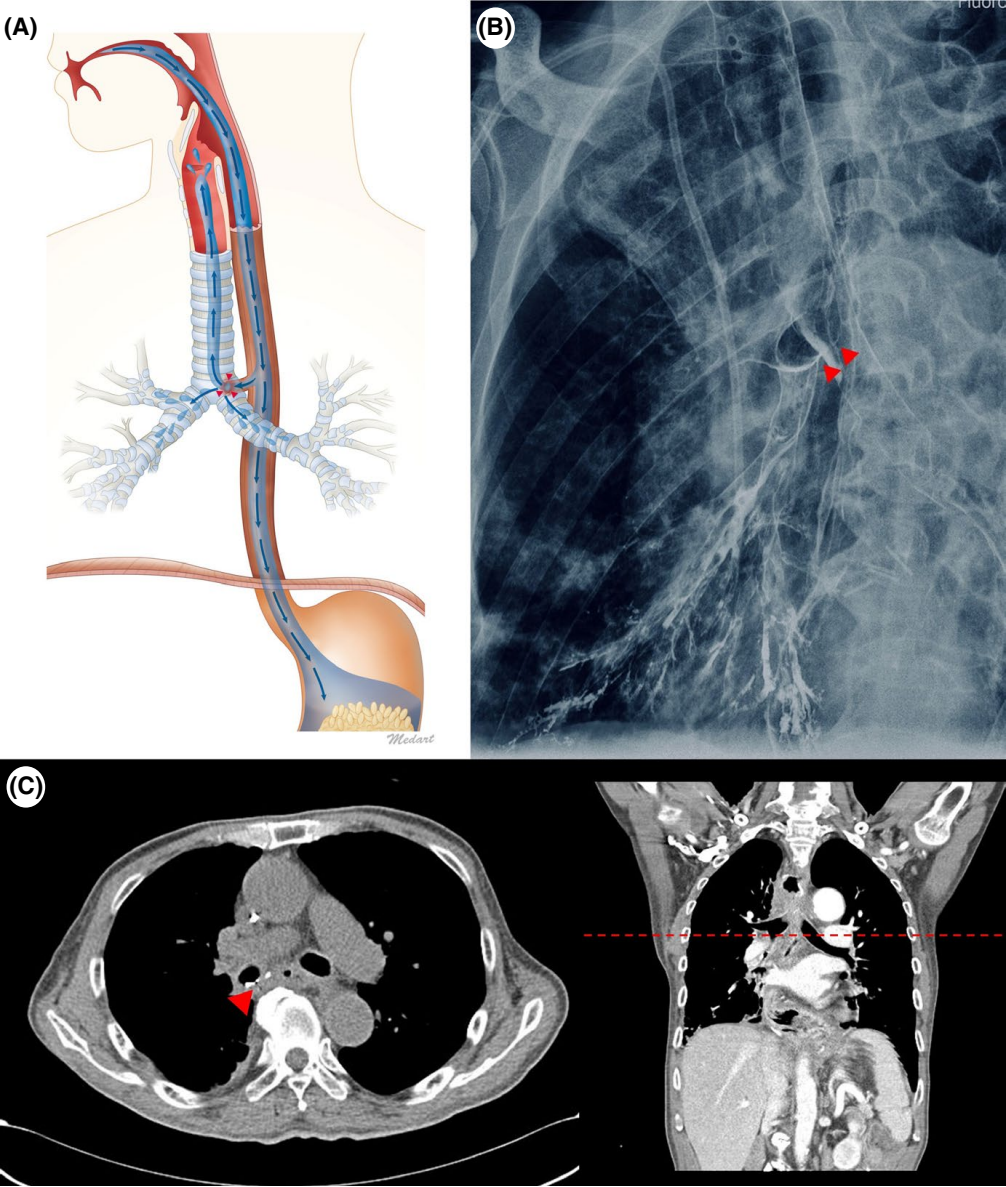
A) Figure showing that the swallowed barium into the airway system pass through small fistula (arrowhead; A), and peribronchial barium infiltration and upward reflux of barium from the trachea to the pharyngeal level (blue arrow; B) Chest X‐ray showing peribronchial barium infiltration and presence of a small fistula (arrowhead; B). C) A computed tomography confirming the presence of a small fistula (arrowhead; C)

The patient was asked to swallow barium liquid via a cup. After swallowing, no direct aspiration was observed. He suddenly developed paroxysmal coughing and the swallowed barium refluxed in the reverse direction, from the trachea to the pharyngeal level. Scanning down to the esophagus showed barium entry into the bronchus via a fistula (Video S1) and confirmed a bronchoesophageal fistula (Figure 1).^1^, ^2^

## CONFLICT OF INTEREST

None declared.

## AUTHOR CONTRIBUTIONS

YH: wrote the manuscript and edited the video. GP: supervised the writing of the manuscript. IS: participated in the procedure. All authors read and approved the final manuscript.

## ETHICAL APPROVAL

This case report was approved by the institutional review board (HC19ZESE0028).

## Supporting information

Video S1
